# Screening of Honey Bee Pathogens in the Czech Republic and Their Prevalence in Various Habitats

**DOI:** 10.3390/insects12121051

**Published:** 2021-11-24

**Authors:** Petr Mráz, Marian Hýbl, Marek Kopecký, Andrea Bohatá, Irena Hoštičková, Jan Šipoš, Kateřina Vočadlová, Vladislav Čurn

**Affiliations:** 1 Faculty of Agriculture, University of South Bohemia in Ceske Budejovice, Studentska 1668, 370 05 Ceske Budejovice, Czech Republic; mario.eko@seznam.cz (M.H.); mkopecky@zf.jcu.cz (M.K.); bohata@zf.jcu.cz (A.B.); jelini00@zf.jcu.cz (I.H.); katerina.vocadlova@gmail.com (K.V.); curn@zf.jcu.cz (V.Č.); 2 Faculty of Agronomy, Mendel University in Brno, Zemedelska 1, 613 00 Brno, Czech Republic; jan.sipos@mendelu.cz

**Keywords:** *Apis mellifera*, deformed wing virus, screening, trypanosomatids

## Abstract

**Simple Summary:**

Worldwide, mass losses of honey bee colonies are being observed more frequently in recent times. Except for the overuse of pesticides, one of the main reasons for high honey bee colony collapse is diseases. For this reason, nationwide screening of common pathogens involving viruses, bacterial, fungal, and protozoa pathogens was performed in three different types of habitat including agroecosystems, towns, and national parks. The most frequent eukaryotic pathogens were Trypanosomatids and *N. ceranae* and in the case of viruses DWV-A and ABPV. In addition, the association between the occurrence of particular pathogens and winter colony losses was found. Although the differences in mortality between individual habitats were not significant, results of this study suggest a significant correlation between DWV-B and DWC-C occurrence and mortality of bee colonies, despite their relatively low occurrence.

**Abstract:**

Western honey bee (*Apis mellifera*) is one of the most important pollinators in the world. Thus, a recent honey bee health decline and frequent honey bee mass losses have drawn attention and concern. Honey bee fitness is primarily reduced by pathogens, parasites, and viral load, exposure to pesticides and their residues, and inadequate nutrition from both the quality and amount of food resources. This study evaluated the prevalence of the most common honey bee pathogens and viruses in different habitats across the Czech Republic. The agroecosystems, urban ecosystems, and national park were chosen for sampling from 250 colonies in 50 apiaries. Surprisingly, the most prevalent honey bee pathogens belong to the family Trypanosomatidae including *Lotmaria passim* and *Crithidia mellificae*. As expected, the most prevalent viruses were DWV, followed by ABPV. Additionally, the occurrence of DWV-B and DWV-C were correlated with honey bee colony mortality. From the habitat point of view, most pathogens occurred in the town habitat, less in the agroecosystem and least in the national park. The opposite trend was observed in the occurrence of viruses. However, the prevalence of viruses was not affected by habitat.

## 1. Introduction

The western honey bee (*Apis mellifera*) is one of the most important pollinators of many agricultural crops and wild plants worldwide. Overall, annual economic evaluation of the pollination service was quantified in 2005 to 153 billion euros, representing a yield of about 10% of global agriculture production [1]. Considering the ecological and economical importance of pollination, the widespread honey bee colony losses are a worrying phenomenon [2]. Researchers have found many factors that are a potential cause of honey bee collapse including viral [3], fungal [4], and bacterial diseases [5] together with the use of pesticides [6]. Other factors leading to the collapse of honey bee colonies are parasites, chemical treatments (amitraz, tau-fluvalinate, coumaphos, antibiotics), nutritional stress (pollen monodiete), and others [7,8]. Some stressors act synergistically such as *Nosema apis* and some pesticides [9]. The collapse of honey bee colonies is thus probably caused by combinations of multiple factors. Therefore, it is necessary to look at and deal with the health of honey bee colonies comprehensively [10].

Recently, however, viral diseases have largely contributed to bee colony losses. The most common and most dangerous virus is a deformed wing virus (DWV). This single-stranded RNA virus is a member of *Iflaviridae* [11] and creates highly genetically heterogeneous forms known as quasispecies, which can exist as several master variants [12]. One of them is type A (DWV-A), which has been attributed to the global decline in honey bees [13,14,15]. Another variant is type B (DWV-B), known as Varroa destructor virus-1 (VDV-1) [16,17], since it was isolated from the Varroa mite for the first time [18]. The third master variant is type C (DWV-C). However, its impact on honey bees is still unclear [12]. Other common honey bee viruses are slow bee paralysis virus (SBPV), acute bee paralysis virus (ABPV), chronic bee paralysis virus (CBPV), black queen cell virus (BQCV), sacbrood virus (SBV), Lake Sinai virus (LSV), and Macula-like virus (MLV) [19]. Their increasing distribution is mainly due to the ubiquity of the *Varroa destructor* mite, which serves as a vector and transmits viruses [10], both directly on honey bees and indirectly on other insect pollinators [20].

Another dangerous pathogen is the bacteria *Paenibacillus larvae* causing the disease called American foulbrood (AFB). Several genotypes (ERIC I-V) of this bacteria are known, and each has its specific properties such as virulence or distribution area [21]. American foulbrood is one of the most infectious honeybee diseases spread worldwide [22]. In some countries (USA, Canada, Argentina), it is allowed to use antibiotics against AFB. However, antibiotic treatment can only mitigate the symptoms but not eliminate the disease. Moreover, the antibiotics leave residues in the honey and their use in beekeeping is prohibited in many countries [23]. Given that the spores of this bacterium are very resilient and remain viable for more than 35 years, the only effective provision against the spread of *P. larvae* is to burn the infected hives together with combustible beekeeping equipment. It is essential to monitor infected habitats and their surroundings for a long time [5].

The bacterium *Melissococcus plutonius*, the causal agent for European foulbrood, has a similar infection course and method of control. It often appears together with other bacteria, so-called secondary invaders. This pathogen causes great problems, especially in the UK and Switzerland [24,25]. However, *M. plutonius* has been recorded in the Czech Republic in 2015 after a long time [26].

Important parasites are also pathogenic fungi *Nosema apis* and *Nosema ceranae,* which cause disease of the digestive tract of adult honey bees. At present, this disease is considered one of the main causes of the collapse of honey bee colonies during the winter period [10,27].

So far, less attention has been drawn to fungal diseases such as chalkbrood disease caused by entomopathogenic fungus *Ascosphaera apis* [28]. It causes mummification of bee larvae in the hive, resulting in weakening the colony and increasing susceptibility to other pathogens. Under suitable environmental conditions, the reproductive potential of the pathogen increases [29]. In some cases, it can even cause the death of bee colonies [30]. In addition, its worldwide distribution and its frequent occurrence make it an economically significant disease on a global scale [31,32].

Recently, of concern is also an infection by parasitic protozoa *Crithidia mellificae* and *Lotmaria passim* belonging to the order Trypanosomatida [33], which were previously considered relatively harmless [34]. However, it turns out that they can cause significant losses of honey bee colonies, especially with co-infection with *Nosema ceranae* [35,36,37]. Castelli et al. [38] also reported an association between the infected colonies and higher level of *V. destructor* infestation. Furthermore, honey bees have a highly conserved and specialized intestinal microbiome [39] that might be disrupted by trypanosomatids [40]. *L. passim* species has only recently been described [33] and now represents the dominant trypanosomatids species [37], which has already been detected in the Czech Republic [40].

All of the above-mentioned pathogens contribute to the deaths of honey bee colonies. In particular, they have a significant negative effect on the bees’ winter generation, which, due to stronger immunity and longevity, ensures the survival of honey bee colonies during winter. However, since the winter generation of bees is weakened, the length of their lives is significantly reduced, which might subsequently lead to honey bee colony losses [41].

To inhibit pathogens within the congenital and social immunity and for the proper development of honey bee brood, the quality of honey bee nutrition represented by pollen is crucial. In particular, its diverse composition with a broader range of biologically active substances significantly contributes to strengthening the bee detoxification capacity [42], immunity, and resistance to overcome some diseases [43] or viral infections [44]. In contrast, the low diversity of food resources can cause malnutrition and, together with the cocktail of pesticides applicable on the fields, can shorten the life of the winter generation of bees. This can disrupt the immune response of bees, which are then more susceptible to pathogens, parasites, and other stressors. This situation occurs more often in intensively cultivated agricultural areas where a significant change in the landscape has been made, leading to a reduction in biodiversity [45]. Very specific are urban areas, which have recently become increasingly popular for beekeeping. These are mainly characterized by a built-up area and high human disturbances. Nevertheless, urban areas also contain parks, gardens, and other seminatural areas, which provide honey bees with continual nectar and pollen flow [46]. Protected areas are represented by a less anthropogenically influenced landscape characterized by a high diversity of vegetation providing rich food resources and a low level of chemical contamination [47].

This study aims to evaluate the prevalence of the main honey bee pathogens in the Czech Republic, depending on different types of habitats representing various anthropogenic burdens as well as to determine the possible impact of individual pathogens and their co-infection on the honey bee colony losses during the winter period.

## 2. Materials and Methods

### 2.1. Sampling

Samplings were carried out from selected apiaries placed in different landscapes across the Czech Republic in the fall of 2019. Agroecosystems, urban ecosystems, and national parks were chosen concerning different urban burdens to sample biological material from 250 hives in 50 apiaries (22 apiaries in agroecosystems, 22 apiaries in urban ecosystems, and six apiaries in the national park). From each apiary, five beehives were randomly chosen. Approximately 50 honey bees were collected from the brood frame of each beehive and immediately frozen on dry ice. The samples were stored at −80 °C until processing. All brood frames from the tested colonies were checked for symptoms of bacterial bee brood diseases. The colony losses were assessed in spring 2020 (the percentage of collapsed colonies of the whole apiaries).

### 2.2. Characterization of Different Types of Habitat

The town habitat in the Czech Republic involves especially built-up area of towns with houses and factories and is affected by increased industrial contamination and high levels of traffic. Therefore, it represents the highest urban burdens. This habitat also includes town parks and gardens. The agroecosystems are characterized by large areas of fields with agricultural crops, especially monocultures, a high rate of landscape fragmentation and agrochemical contamination. In addition, low diversity of bee food sources as well as short-term availability of food due to intensive agricultural management is typical. National parks, as the most potential honey bee-friendly environment with minimal human disturbance is characterized by flowery meadows, pastures, and forests. Habitat is characterized by an absence of industry, a low degree of landscape fragmentation, and a rich diversity of flowers, which are a good source of food for bees. Agricultural management is possible only through an ecological approach without the use of pesticides.

### 2.3. Sample Preparation and Nucleic Acid Purification

Samples for RNA (detection of DWV-A, DWV-B, DWV-C, BQCV, CBPV, ABPV, SBV, LSV, MLV) and DNA (detection of *Nosema apis*, *Nosema ceranae*, *Paenibacillus larvae*, *Melissococcus plutonius*, *Ascosphaera apis*, *Crithidia mellificae*, *Lotmaria passim*) purification were collected as a bulk of approximately 250 bees from five hives in each location, frozen in dry ice, and stored at −80 °C. After homogenization in liquid nitrogen, aliquotes for separate RNA and DNA purification were made.

According to the manufacturer’s instructions, total RNA was extracted using the TRI Reagent (MRC, Montgomery, OH, USA). Contaminating DNA was removed using the DNA-free TM Kit (Ambion, supplied by ThermoFisher Scientific, Loughborough, UK). BioSpec Nano (Shimadzu, Nakagyo-ku, Kyoto, Japan) was used to quantify RNA (OD260) and to assess sufficient quality (OD260/280 ratio and OD260/230 ratio). cDNA templates were prepared using a Standard Reverse Transcription Protocol (Promega, Madison, WI, USA) and OligodT primer and stored at −20 °C until use.

DNA was extracted using a modified CTAB method. Homogenized tissue was resuspended in CTAB buffer (2% CTAB, 100 mM Tris pH 8.0, 20 mM EDTA pH 7.8, 1.4 M NaCl) with 1% β-mercaptoethanol and incubated at 65 °C for 10 min. The solution was extracted with 500 µL chloroform:isoamylalcohol (24:1) and precipitated in 250 µL of 2-propanol at −20 °C for 30 min. After washing with 1 mL of 70% ethanol, the pellet was resuspended in 150 µL of TE buffer (10 mM Tris pH 8.0, 1 mM EDTA pH 7.8) and stored in 4 °C until use.

### 2.4. PCR Conditions

The RT-PCR (detection of DWV-A, DWV-B, DWV-C, BQCV, CBPV, ABPV, SBV, LSV, MLV) was performed on the QuantStudio™ 6 Flex Real-Time PCR System (Applied Biosystems, supplied by ThermoFisher scientific, Loughborough, UK) using Power SYBR^®^ Green PCR Master Mix (Applied Biosystems, supplied by ThermoFisher Scientific, Loughborough, UK) in a 96-well reaction plate using parameters recommended by the manufacturer (2 min at 50 °C, 10 min at 95 °C, and 40 cycles of 15 s 95 °C, 1 min of 60 °C, 15 s at 95 °C, 1 min at 60 °C, and 15 s at 95 °C). The no-template controls were included. Positive samples were considered a true positive using a Ct cutoff of 36 cycles. The specificity of amplification was determined by dissociation curve analyses and sequencing of randomly selected positive samples. The sequence of the primer, orientation, annealing temperature, and references are shown in Table 1.

The PCR (detection of *Nosema apis*, *Nosema ceranae*, *Paenibacillus larvae*, *Melissococcus plutonius*, *Ascosphaera apis*, *Crithidia mellificae*, *Lotmaria passim*) was performed on the Eppendorf Mastercycler PRO system (Eppendorf, Hamburg, DE) in 25 µL volume containing 1× PPP Master Mix (Top-Bio, Vestec, Czech Republic), 10 pmol each forward and backward primer, and 2 µL of DNA template using the following cycling conditions: denaturation at 95 °C for 5 min, 40 cycles of 30 s 95 °C, 45 s of TA, 1 min at 72 °C; and a final extension at 72 °C for 10 min. PCR products were visualized by 1.5% agarose gel electrophoresis and stained with ethidium bromide solution (Merck Life Science, Darmstadt, Germany). The specificity of amplification was determined by sequencing randomly selected positive samples. The sequence of the primer, orientation, annealing temperature, and references are shown in Table 1.

### 2.5. Statistical Analysis

To evaluate whether pathogen occurrence and species richness differ among honey bee colonies and habitat types, we used separate generalized liner mixed-effects models (GLMM) [59]. In the case when species richness was used as dependent variable, GLMM with a Gaussian error distribution was used. When the pathogen occurrence or honey bee mortality rate was used as the dependent variable, binomial error distribution with logit link function was used. In each model, we specify habitat types and pathogen species as fixed factors and the owner of the honey bee colony was used as a factor with a random intercept effect. To compare the means within a particular fixed factor, the Tukey multiple comparison test with Bonferroni adjustment of *p*-values was used. Data were analyzed in the R program (R Development Core Team 2020).

To visualize and test the association between the mortality rate of honey bees and species composition of pathogens, partial canonical correspondence analysis (pCCA) was used with the habitat type as the covariable. We used this type of covariable to eliminate the possible confounding effect of habitat type on the mortality of honey bees regardless of the pathogen species composition. The significance of the canonical axis was tested with a restricted Monte Carlo permutation test for the time series with 2000 permutations. All ordination analyses were conducted by the statistical software CANOCO, v. 5 [60].

## 3. Results

**Figure 1:**
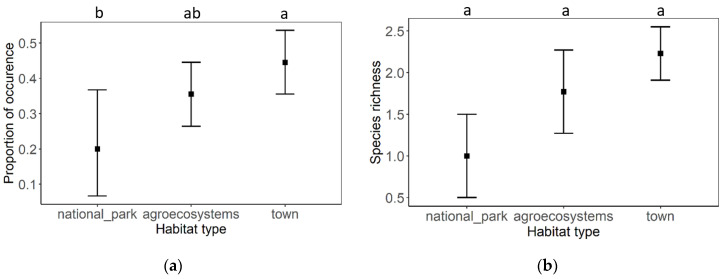
(**a**) The proportion of eukaryotic pathogen occurrence in different types of habitats and (**b**) the proportion of eukaryotic pathogen richness in different types of habitats. Black squares represent means and the error bars represent 95% confidence intervals. Significant differences (<0.05) are indicated by different letters.

The proportion of eukaryotic pathogen occurrence significantly differs between town habitat and national park, whereas the lowest rate of pathogen occurrence has been observed in the national park and the highest in the towns. A moderate rate of pathogen burden has been observed in agroecosystems. However, this habitat did not differ significantly between urban areas or national parks (Figure 1a, Table 2). The species richness of eukaryotic honey bee pathogens did not significantly differ between the tested habitats (Figure 1b, Table 2).

**Figure 2:**
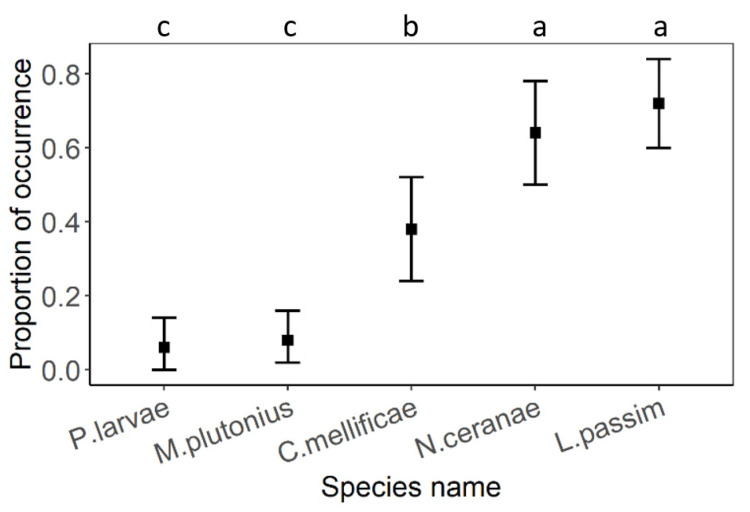
The comparison of proportion of eukaryotic pathogen occurrence regardless of habitat. Black squares represent means and the error bars represent 95% confidence intervals. Significant differences (<0.05) are indicated by different letters.

In all types of habitat, the same species of eukaryotic pathogens dominated. In all cases, the most dominant species were *L. passim* and *N. ceranae*, followed by *C. mellificae*, and the lowest occurrence rate had *M. plutonius* and *P. larvae*. No clinical symptoms of bacterial brood diseases were observed. In contrast, *A. apis* and *N. apis* were not detected at all (Figure 2).

**Figure 3:**
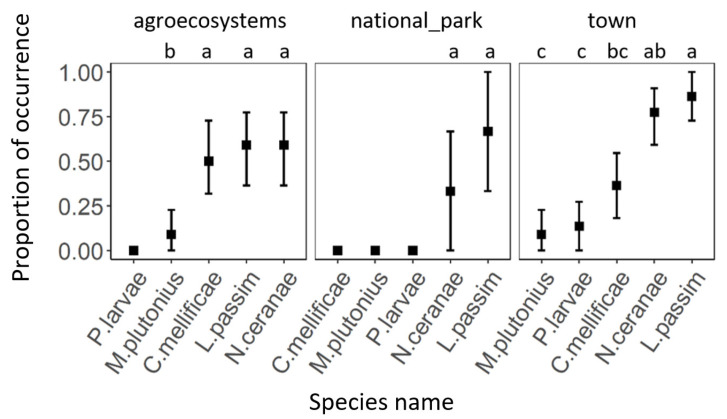
The comparison of proportion of eukaryotic pathogen occurrence within each habitat type. Black squares represent means and the error bars represent 95% confidence intervals. Significant differences (<0.05) are indicated by different letters.

In the case of individual habitats, all five tested pathogens were detected in a town habitat. The most prevalent pathogens were *L. passim* and *N. ceranae*, followed by *C. mellificae*. Bacteria *P. larvae* and *M. plutonius* only had a low prevalence. The most dominated species in the agroecosystems were *N. ceranae*, *L. passim*, and *C. mellificae*. *M. plutonius* occurred significantly less and *P. larvae* were not detected at all. In the case of national parks, only *L. passim* and *N. ceranae* were detected (Figure 3).

**Figure 4:**
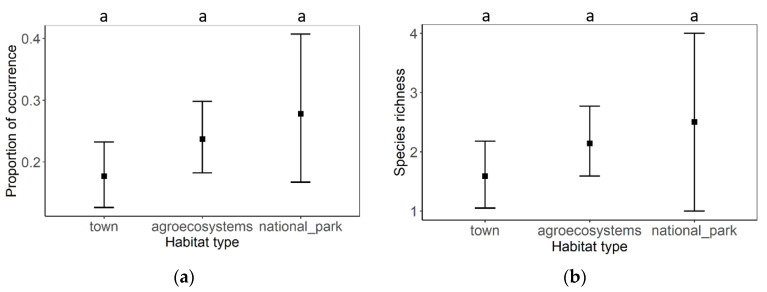
(**a**) The proportion of viral pathogens occurrence in different types of habitats and (**b**) comparison of species richness of viral pathogens between different types of habitats. Black squares represent means and the error bars represent 95% confidence intervals. Significant differences (<0.05) are indicated by different letters.

**Figure 5:**
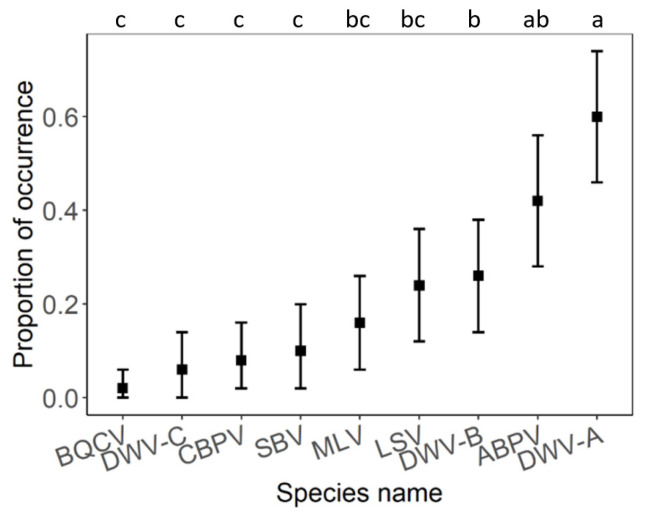
The comparison of proportion of viral pathogen occurrence regardless of habitat. Black squares represent means and the error bars represent 95% confidence intervals. Significant differences (<0.05) are indicated by different letters.

**Figure 6:**
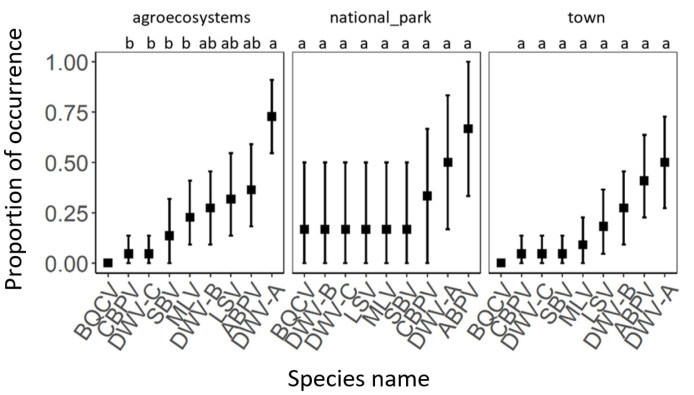
The comparison of proportion of viral pathogen occurrence within each habitat type. Black squares represent means and the error bars represent 95% confidence intervals. Significant differences (<0.05) are indicated by different letters.

Viral pathogen occurrence and species richness did not significantly differ between individual habitats (Figure 4 and Table 2). Generally, the most abundant viruses were DWV-A and ABPV, followed by DWV-B and LSV. Less frequent viruses were MLV, SBV, CBPV, DWV-C, and BQCV (Figure 5). A similar pattern was observed in all types of habitats. Only DWV-A dominated in the agroecosystems (Figure 6).

**Figure 7:**
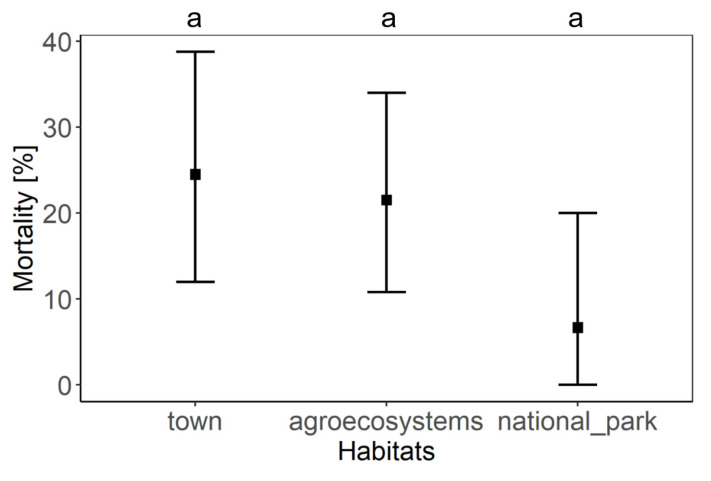
The comparison of honey bee winter mortality rate according to type of habitat. Black squares represent means and the error bars represent 95% confidence intervals.

Differences winter mortality rates in honey bee colonies between habitats were not statistically significant (Figure 7) due to a small number of samples from national parks and high confidence interval from the data. However, the average winter mortality in town (24.51%) and agroecosystem (21.50%) habitats were twice as high as in national parks (11.11%).

**Figure 8:**
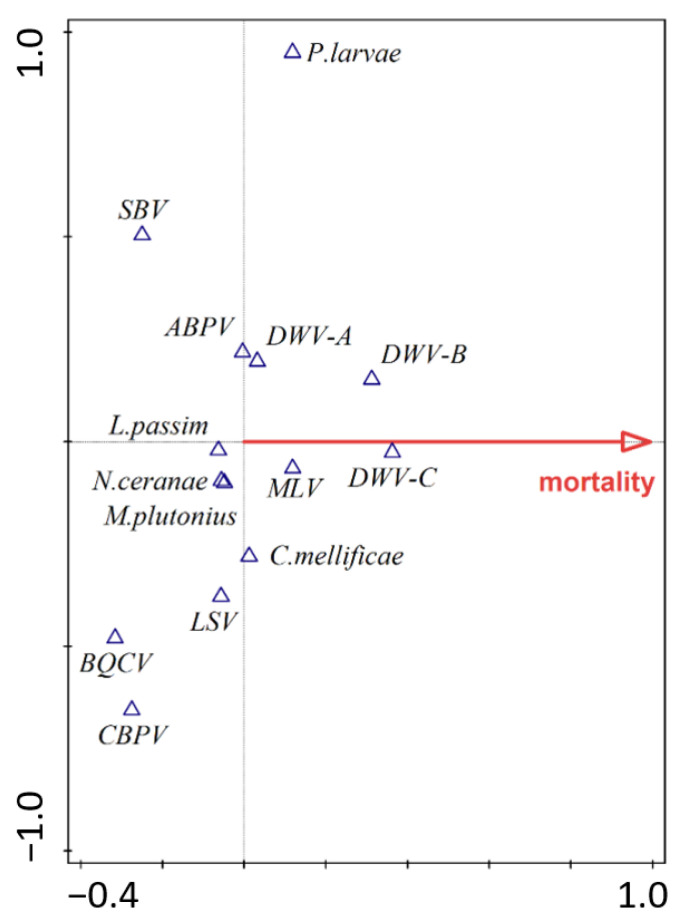
Partial canonical correspondence analysis biplot with the habitat type as a covariable showing the strength of the association of individual pathogens with the mortality rate.

Based on the results of pCCA species, structures of all pathogens (i.e., species composition and their abundances) were significantly associated with honey bee mortality (pseudo-F = 1.8, *p* = 0.053, test of all canonical axes, R^2^ = 3.73%). In the separate pCCA analyses evaluating association only between viruses and honey bee mortality, we found that the assemblage composed only with viruses (pseudo-F = 2.2, *p* = 0.037, test of all canonical axes, R^2^ = 5.28%) had a closer relationship to mortality than the assemblage composed only with eukaryotes (pseudo-F = 0.3, *p* = 0.881, test of all canonical axes, R^2^ = 0.80%). The pCCA diagram revealed that the closest association with honey bee mortality was shown by DWV-C and DWV-B viruses (Figure 8).

## 4. Discussion

In the study, the prevalence of several honey bee pathogens was detected including viruses, fungal, protozoa, and bacterial pathogens on different types of habitats. The most frequently detected pathogens belonged to the family Trypanosomatida, in particular, *Lotmaria passim* (72%) and *Crithidia mellificae* (38%). Both protozoa significantly shorten the life of bees and are therefore thought to cause significant bee colony losses [61]. Of even more concern is that trypanosomatids affect the composition of the symbiotic bacterial taxa of bees [40]. However, little is known about the full extent of the harmfulness and mechanism of pathogenesis of these two pathogens [38,62]. Other studies have shown an even higher risk of trypanosomatids when co-infected with *N. ceranae* [35,36,37]. In addition, it led to a reduction in immune gene expression [37]. The high incidence of trypanosomatids is similar in other European countries [36,62].

The prevalent pathogen is also *Nosema ceranae* (64%), often associated with colony losses, especially in Mediterranean areas [4,63,64]. However, its occurrence has also been recorded in the temperate zone to a lesser extent [10] and with less impact [65,66]. In this study, *N. ceranae* has not been significantly associated with colony losses (Figure 8). This pathogen occurred independently of the habitat type observed. On the other hand, *Nosema apis* was not detected at all. The decline in *N. apis* and the spread of *N. ceranae* is a well-known and long-lasting trend taking place globally [67,68,69,70,71]. However, the complete absence of *N. apis* in the nationwide screening is a novelty. We attribute this to the displacement of the more aggressive *N. ceranae* due to its higher virulence [68,69]. *Ascosphaera apis* was also not been detected. It is an opportunistic pathogen that occurs in the colony, especially in stressful situations such as thermal discomfort [29]. Higher prevalence was recorded in humid areas, and, for example, in China [72] and northern Thailand [30], the fungal pathogen causes great damage.

Bacterial diseases occurred only to a lesser extent and only in urban areas *(P. larvae* and *M. plutonius*) and agroecosystems (*M. plutonius*). They did not occur in the national parks at all. *P. larvae* commonly occurs across the whole Czech Republic, especially in Moravia, and the dominant genotype is ERIC II (80.4%) over ERIC I (19.4%) [73]. The outbreak of European foulbrood caused by *M. plutonius* was observed in 2015 after 40 years in the Czech Republic. Since then, the occurrence persists, but with a very low prevalence [74]. In contrast, in some countries such as England [75], France [76], and Switzerland [77], bacterial disease very often occurs. These two bacterial diseases are very infectious and can cause great economic losses. Therefore, the government often monitors its prevalence, and in many cases, there is an effort to eliminate them through strict rules.

In the case of viral diseases, at least one of the tested honey bee viruses were detected in 74% of cases, while two or more viruses were present in one-third of the tested apiaries. The most prevalent honey bee virus was the deformed wing virus (DWV). There are multiple variants of DWV that include type A [11], type B (Varroa destructor virus-1 (VDV-1) [14,18], and type C [12]. These variants have a different impact on honey bee colonies, and their virulence is not clear. Whereas some studies claim DWV-A has higher virulence [16,78,79], other studies claim DWV-B has the same or even higher virulence [17,80,81,82]. Since the variant DWV-B can replicate in Varroa mites, the viral load is usually higher in honey bee tissues than in other DWV variants [78,83]. DWV-C is associated with DWV-A and has been indicated as a contributing factor in overwintering losses of honey bee colonies [78,79]. Our study reports DWV-A as the most frequent variant (60%) in the Czech Republic (Figure 5). Surprisingly, similar results where variant DWV-A dominated have been reported from the USA [79,83], whereas variant B dominated in Europe [78,80,84]. However, despite their low prevalence, only DWV-B (26%) and C (6%) variants were significantly associated with the overwintering losses (Figure 8). Other authors have also concluded that these variants are associated with winter colony losses [17,85].

The second most prevalent virus was ABPV, which was detected in half of the tested colonies. This virus has commonly been detected in Germany [10], the USA [3], Switzerland [86], and Belgium [87] and its co-infection with DWV is attributed to overwintering losses [10]. The LSV (24%) virus is also a major concern, especially in the USA [88]. However, its prevalence is also high in Europe [36]. One of the recently identified honey bee viruses is MLV (16%), which is associated with the mite *V. destructor* [89]. However, its virulence and impact on honey bees are still unclear [90]. Its high prevalence has been observed in France [89], Belgium [36], and Syria [91]. The occurrence of SBV (10%), CBPV (8%), and BQVC (2%) was only minor, especially in urban areas and agroecosystems. The presences of these viruses were not significantly related to the decline of honey bee colonies in the Czech Republic.

The lowest occurrence of eukaryotic pathogens was detected in the national parks, higher occurrence in the agroecosystems, and the highest occurrence in town habitats (Figure 1). This probably corresponds with a high density of bee colonies in the landscape [92] because the number of bee colonies per km^2^ in the Czech Republic is one of the highest in the world (>8 honey bee colonies/km^2^) [93]. According to these results, Taric [94] also found a higher parasitic burden in commercially kept colonies than traditionally kept colonies, which are mostly situated in natural areas. The richness of individual pathogens was in the same trend, where only two eukaryotic pathogens were present in the national parks. At the same time, four of them occurred in the agroecosystems and five in the towns.

The opposite trend was observed for viruses. All nine tested viruses were present in the national parks, while in agroecosystems and towns, there were eight species. However, these differences were not statistically significant. The study shows that the occurrence of honey bee pathogens, and especially viruses, did not differ between the tested habitats. In addition, the viruses also spread quickly among other species of wild pollinators, which can cause problems with species composition and affect trophic bonds and ecosystem stability [20,84,95].

Differences in the mortality between habitats were not statistically significant. The results were not significant probably due to the low number of samples from the national parks. One of the reasons for colony mortality in national parks is probably due to the high prevalence of viruses as in other habitats (DWV-B and DWV-C), which were associated with colony mortality. The next issue is the trading of bee queens or whole colonies and the migratory management of colonies [96]. This is connected with colony density, which is usually lower in natural parks. This might be another reason for lower honey bee eukaryotic pathogen occurrence in natural parks. At localities with a high bee density, bee colonies cannot avoid sharing food resources, which represent hotspots of infections [97].

## 5. Conclusions

The most prevalent eukaryotic pathogens in the population of *A. mellifera* in the Czech Republic were *L. passim* and *N. ceranae*, followed by *C. mellificae*. This trend was valid in all types of monitored habitats. In contrast, *P. larvae* and *M. plutonius* were detected only sporadically. *N. apis* and *A. apis* were not detected at all.

The most prevalent viruses were DWV-A and ABPV in all types of tested habitats. On the other hand, BCQV, SBV, and DWV-C were the least prevalent, except in national parks, where the occurrence of all the monitored viruses was relatively uniform.

Of all the monitored eukaryotic and viral pathogens, only DWV-C and DWV-B were significantly associated with colony mortality.

## Figures and Tables

**Table 1:** Primers for PCR analysis.

Gene	Sequences 5′-3′	TA [°C]	Reference
***Nosema apis***	F: GGGGGCATGTCTTTGACGTACTATGTA R: GGGGGGCGTTTAAAATGTGAAACAACTATG	62	[48]
***Nosema ceranae***	F: CGGCGACGATGTGATATGAAAATATTAA R: CCCGGTCATTCTCAAACAAAAAACCG	62	[48]
***Paenibacillus larvae***	F: GCTCTGTTGCCAAGGAAGAA R: AGGCGGAATGCTTACTGTGT	55	[49]
***Melissococcus plutonius***	F: GAAGAGGAGTTAAAAGGCGC R: TTATCTCTAAGGCGTTCAAAGG	55	[50]
***Ascosphaera apis***	F: TGTGTCTGTGCGGCTAGGTG R: GCTAGCCAGGGGGGAACTAA	60	[51]
***Crithidia mellificae***	F: AGTTTGAGCTGTTGGATTTGTT R: AACCTATTACAGGCACAGTTGC	56	[52]
***Lotmaria passim***	F: TGACTTGAATTAGCAAGCATGGGATAACA R: CCTTTAGGCTACCGTTTCGGCTTTTGTTGGT	60	[53]
**DWV-A**	F: CGTCGGCCTATCAAAG R: CTTTTCTAATTCAACTTCACC	60	[54]
**DWV-B**	F: GCCCTGTTCAAGAACATG R: CTTTTCTAATTCAACTTCACC	60	[54]
**DWV-C**	F: TACTAGTGCTGGTTTTCCTTT R: ATAAGTTGCGTGGTTGAC	60	[54]
**BQCV**	F: GGACGAAAGGAAGCCTAAAC R: ACTAGGAAGAGACTTGCACC	48	[48]
**CBPV**	F: AACCTGCCTCAACACAGGCAAC R: ACATCTCTTCTTCGGTGTCAGCC	60	[55]
**ABPV**	F: TGAGAACACCTGTAATGTGG R: ACCAGAGGGTTGACTGTGTG	48	[56]
**SBV**	F: GGATGAAAGGAAATTACCAG R: CCACTAGGTGATCCACACT	48	[56]
**LSV**	F: CKTGCGGNCCTCATTTCTTCATGTC R: CATGAATCCAAKGTCAAAGGTRTCGT	60	[57]
**MLV**	F: ATCCCTTTTCAGTTCGCT R: AGAAGAGACTTCAAGGAC	60	[58]

**Table 2:** The results of the analysis of deviance (likelihood-ratio test) testing the partial effect of habitat type and pathogen species identity on the species richness and occurrence of pathogens in the honey bee colonies. Likelihood-ratio analysis testing of whether the Akaike information criterion (AIC) of the full model significantly increased after a particular explanatory variable was excluded from the model.

	Df.	AIC	LRT	Pr (Chi)
Dependent variable: species occurrence				
Full model		243.68		
Eukaryote	4	332.25	96.570	<0.0001
Habitat	2	246.76	7.081	0.02899
Full model		398.26		
Virus	9	494.96	114.695	<0.0001
Habitat	2	396.69	2.423	0.2977
Dependent variable: number of eukaryotic species				
Full model		156.78		
Habitat	2	157.23	4.453	0.107
Dependent variable: number of virus types				
Full model		181.88		
Habitat	2	180.52	2.642	0.267

## Data Availability

The study did not report any data.
